# Smoking status and cause-specific discontinuation of tumour necrosis factor inhibitors in axial spondyloarthritis

**DOI:** 10.1186/s13075-019-1958-z

**Published:** 2019-07-22

**Authors:** Sizheng Steven Zhao, Kazuki Yoshida, Gareth T. Jones, David M. Hughes, Stephen J. Duffield, Sara K. Tedeschi, Houchen Lyu, Robert J. Moots, Daniel H. Solomon, Nicola J. Goodson

**Affiliations:** 1 Musculoskeletal Biology I, Institute of Ageing and Chronic Disease, University of Liverpool, Liverpool, UK; 2 Department of Academic Rheumatology, Aintree University Hospital, Liverpool, UK; 3 Division of Rheumatology, Immunology and Allergy, Brigham and Women’s Hospital, Boston, MA USA; 4 Departments of Epidemiology and Biostatistics, Harvard T.H. Chan School of Public Health, Boston, MA USA; 5 Epidemiology Group, School of Medicine, Medical Sciences and Nutrition, University of Aberdeen, Aberdeen, UK; 6 Aberdeen Centre for Arthritis and Musculoskeletal Health, University of Aberdeen, Aberdeen, UK; 7 Department of Biostatistics, Institute of Translational Medicine, University of Liverpool, Liverpool, UK; 8 Department of Medicine, Harvard Medical School, Boston, MA USA; 9 Department of Orthopaedics, General Hospital of Chinese PLA, Beijing, China; 10 Division of Pharmacoepidemiology and Pharmacoeconomics, Department of Medicine, Brigham and Women’s Hospital and Harvard Medical School, Boston, MA USA

**Keywords:** Axial spondyloarthritis, Ankylosing spondylitis, TNF inhibitor, Biologic DMARDs, Persistence, Effectiveness, Response, Discontinuation, Marginal structural model

## Abstract

**Background:**

The impact of smoking on TNF inhibition (TNFi) therapy is unclear. We examined the effect of smoking on all-cause and cause-specific TNFi discontinuation in axial spondyloarthritis (axSpA).

**Methods:**

We used longitudinal data from the British Society for Rheumatology Biologics Register for Ankylosing Spondylitis (BSRBR-AS). Patients fulfilling the ASAS criteria for axSpA, who started their first TNFi, were eligible for analysis. Inverse-probability weights were used to balance differences in baseline disease severity and other confounders. We used marginal structural Cox proportional hazard models to estimate hazard ratios (HR) for TNFi discontinuation according to smoking status. In analyses of cause-specific discontinuation, competing risk events were considered as censoring, using inverse-probability weights.

**Results:**

A total of 758 participants were included in the analysis (66% male, mean age 45 years), providing 954 patient-years of follow-up. TNFi was discontinued in 174 (23%) patients, among whom 26% stopped due to infections, 20% due to other adverse events and 44% due to inefficacy or other reasons. Thirty-four percent were current smokers and 30% ex-smokers. Compared to never smokers, current smokers’ risk of TNFi discontinuation was HR 0.79 (95%CI 0.53 to 1.20) and ex-smokers HR 0.68 (95%CI 0.45 to 1.04). Our data did not show evidence that current smoking influenced discontinuation due to infections (HR 0.79, 95%CI 0.40 to 1.54), other adverse events (HR 0.86, 95%CI 0.41 to 1.78) or inefficacy/other causes (HR 1.44, 95%CI 0.86 to 2.41).

**Conclusion:**

Baseline smoking status did not impact TNFi discontinuation in this UK cohort of axSpA participants.

**Electronic supplementary material:**

The online version of this article (10.1186/s13075-019-1958-z) contains supplementary material, which is available to authorized users.

## Background

The aetiological and prognostic roles of cigarette smoking in rheumatoid arthritis are well-established, but its role in axial spondyloarthritis (axSpA) remains unclear. Clinically meaningful differences in response to TNF inhibitors (TNFi) have not been demonstrated in axSpA when continuous outcomes are used, such as the Bath Ankylosing Spondylitis Disease Activity Index (BASDAI) [1–3]. In contrast, analyses using binary response variables, such as BASDAI50 (50% reduction), found current smokers to have significantly lower odds of TNFi response compared to never smokers [1, 4]. Since all causes of discontinuation are often counted as non-response, increased drug discontinuation in smokers may explain this discrepancy.

One prior study reporting increased risk of treatment discontinuation in smokers did not adjust for socioeconomic status, comorbidities or baseline disease severity [4]. Since these are potential confounders or known predictors of TNFi discontinuation [5], limited conclusions can be drawn about the independent relationship between smoking and TNFi discontinuation.

Patients stop TNFi for several reasons, including inefficacy, adverse events or other reasons, such as patients’ social circumstances. Infections are adverse events of special interest since smoking is a substantial risk factor for bacterial and viral infections [6]. Whether the effect of smoking differs according to each cause of TNFi discontinuation is not known. Once treatment is stopped for one reason (e.g. adverse events), the individual can no longer be at risk of other causes of discontinuation (e.g. inefficacy) in that treatment episode. When an event either precludes observation of the outcome of interest or modifies its probability, “competing risks” is present [7]. When competing risks are not rare, i.e. > 5% of the population, traditional survival analyses should not be used and additional methodological considerations are needed to appropriately understand a potential association between smoking and TNFi discontinuation.

The aim of the current analyses was to examine the impact of smoking on all-cause and cause-specific TNFi discontinuation in patients with axSpA, using marginal structural models to address the above methodological issues.

## Methods

### Study design and population

We used data from the British Society for Rheumatology Biologics Register for Ankylosing Spondylitis (BSRBR-AS), a UK-wide prospective cohort study of patients fulfilling the Assessment of SpondyloArthritis international Society (ASAS) criteria for axSpA [8]. The current analysis focused on new users of TNFi (Humira, Enbrel/Benepali, Cimzia and Simponi) from December 2012 to June 2017. Patients were assessed at baseline, 3, 6 and 12 months and annually thereafter, with additional follow-ups permitted in the interim. TNFi start and stop dates were ascertained from their medical notes. Participants who completed a valid baseline questionnaire (within 1 year before and 7 days after starting TNFi) and recorded smoking status were eligible for analysis.

### Exposure and outcomes

Self-reported smoking status at baseline (current, ex- or never) was used to define the exposure. The outcomes of interest were time to all-cause and cause-specific TNFi discontinuation. Reasons for stopping were categorised in the BSRBR-AS as adverse events, inefficacy, symptom in remission, or other, with accompanying free-text to further elaborate. Each entry was manually reviewed and, where necessary, re-categorised. To allow comparison with existing studies, and so that each specific cause has sufficient events with which to perform the analyses, we grouped causes as (1) infection-related adverse events, (2) all other adverse events, and (3) inefficacy or other reasons (e.g. patient choice and social circumstances). The censoring date was defined as the last study visit or, if no data existed past the baseline visit, as 3 months (the minimum per protocol follow-up interval).

### Covariates

The following covariates were recorded at baseline and chosen a priori for their possible associations with TNFi discontinuation based on prior literature [5]: age, gender, symptom duration, education, elevated baseline CRP (above upper normal limit), classification as ankylosing spondylitis (modified New York criteria), social deprivation (quintiles) [9], alcohol use (current, previous, never), comorbidity (categorised as 0, 1 or ≥ 2 from a list of 13 conditions [8]), body mass index (BMI), TNFi agent and year of TNFi initiation. Patient-reported variables were measured at each follow-up; those known to be associated with TNFi discontinuation include BASDAI, spinal pain, functional impairment (BASFI) and fatigue (Chalder Fatigue Scale). Since these variables capture more than just disease activity, we refer to them collectively as “disease severity” throughout the text.

### Statistical analysis

Baseline participant characteristics were summarised by smoking status. Unadjusted comparison of time to all-cause discontinuation according to smoking status was made using Kaplan-Meier estimators.

Prior studies considered each cause of TNFi discontinuation as censoring [10]. In traditional survival analysis, observation time for each subject is censored when they leave the study or at the end of follow-up. Furthermore, a key assumption of Cox models is that censored patients should be representative of remaining individuals at that time point (i.e., censoring is random). Patients who discontinue treatment are not representative of those who continue; therefore, Cox models cannot be used.

Marginal structural models use inverse-probability weights to account for confounders rather than adjusting for them in conventional outcome regression [11]. This approach is more flexible, allowing us to account for baseline imbalance in characteristics between smoking status, time-varying disease activity, and competing risk events within one model. For all following analyses, differences in baseline covariates between smoking categories were balanced using inverse probability of “treatment” weights (IPTW) [11] to allow unconfounded descriptive comparisons [12]. Causal inference is not straightforward regarding the effect of baseline smoking status on TNFi discontinuation, since we cannot randomly assign an individual to “having smoked for 20 years” at the onset of a hypothetical trial. IPTW provides an approach to estimate causal effects under theoretical ideal conditions and may be advantageous over regression adjustment (see Additional file 1). We derived IPTW using predicted values from a multinomial logistic regression model, using baseline smoking status as the dependent variable and all baseline covariates specified above as independent variables.

We applied baseline time-invariant inverse probability of censoring weights (IPCW) to account for participants excluded from the sample eligible for analysis, such that baseline characteristics of the analysis set resembled the eligible TNFi exposed cohort. These IPCWs were constructed in the same manner as IPTWs, with inclusion/exclusion status as the dependent variable.

We used marginal structural Cox proportional hazards models to estimate hazard ratios of TNFi discontinuation according to baseline smoking status [11, 13]. Parameters were estimated using weighted pooled logistic models that are equivalent to Cox models when using discrete time [14] and allow the use of subject-specific time-varying weights. Time was split into integer months [13].

Censoring becomes non-random when competing risk events are specified as censoring. We therefore calculated time-varying IPCWs, such that censoring becomes random at each time point with respect to baseline characteristics and history of disease activity [15]. For analysis of each specific cause of TNFi discontinuation (e.g. infection), the other competing risk events (e.g. other reasons) were treated as censoring events.

Weighted pooled logistic models included only the outcome, exposure (smoking status) and time, which was included in all models as restricted cubic splines [13]. All weights were “stabilised” to have a mean of 1, allowing the overall sample size to remain unchanged [16]. Further details of the methods are given in [13]. Multiple imputation was used for missing covariates. Analyses were conducted using Stata version 13.

## Results

Among a total of 2420 participants in the BSRBR-AS, 840 commenced their first TNFi within the study period and had smoking status recorded at baseline. Eighty-two participants were excluded because they did not have a valid baseline assessment. Excluded participants had longer symptom duration, more frequently met classification criteria for AS and were more frequently male (full comparison in Additional file 1: Table S1). Seven hundred fifty-eight participants were included in the analysis set, providing 954 patient-years of follow-up. The median follow-up time was 1.0 year (inter-quartile range 0.4 to 2.0 years).

TNFi was discontinued in 174 (23%) patients. The median time to discontinuation was 6 months (inter-quartile range 3 to 11). Among those who stopped TNFi, 26% stopped due to infections, 20% due to other adverse events and 44% due to inefficacy or other reasons, with no differences according to smoking status. Recoding of discontinuation causes is shown in Additional file 1: Table S2.

Baseline characteristics of the analysis cohort are shown in Table 1. Thirty-four percent of patients were current smokers, 30% ex-smokers and 36% never smokers. Current smokers were younger, were more frequently male and showed trends for having higher deprivation and lower educational attainment. Current smokers also more frequently had elevated CRP. The three groups did not differ in age of symptom onset. Current smokers reported worse baseline disease activity and functional impairment. Covariate imbalance was negligible (standardised mean differences < 0.1) after weighting for IPTW (Additional file 1: Figure S1).

**Table 1:** Baseline characteristics and causes of discontinuation among 758 participants, according to smoking status

		Never smoker (*n* = 224)	Ex-smoker (*n* = 177)	Current smoker (*n* = 197)	*P* value
Age, mean (SD) years		43.3 (14.5)	50.0 (12.8)	42.2 (11.8)	*< 0.001*
Male		167 (62%)	149 (65%)	188 (73%)	*0.012*
Meets mNY criteria for AS		164 (61%)	154 (67%)	162 (63%)	0.360
HLA-B27 positive^+^		143 (70%)	125 (76%)	148 (80%)	0.088
Elevated CRP*		150 (57%)	129 (59%)	161 (67%)	*0.043*
Age at symptom onset, median (IQR) years		26.0 (20.0 to 33.0)	26.0 (22.0 to 34.0)	26.0 (21.0 to 33.0)	0.350
Symptom duration, median (IQR) years		12.4 (4.2 to 26.0)	20.7 (10.3 to 32.5)	12.3 (5.5 to 22.9)	*< 0.001*
BMI, mean (SD)		27.9 (6.2)	28.8 (5.1)	27.4 (5.8)	0.060
Quintiles of Index of Multiple Deprivation	1, most deprived	39 (14%)	33 (14%)	85 (33%)	< 0.001**
	2	54 (20%)	30 (13%)	43 (17%)	
	3	52 (19%)	53 (23%)	51 (20%)	
	4	68 (25%)	56 (24%)	47 (18%)	
	5, most affluent	58 (21%)	59 (26%)	30 (12%)	
Highest level of education	Secondary school	72 (27%)	75 (33%)	114 (45%)	*< 0.001*
	Apprenticeship	18 (7%)	24 (10%)	29 (12%)	
	Further education college	83 (31%)	81 (35%)	71 (28%)	
	University degree	70 (26%)	38 (17%)	29 (12%)	
	Further degree	26 (10%)	11 (5%)	9 (4%)	
Alcohol status	Never	31 (11%)	14 (6%)	30 (12%)	*< 0.001*
	Ex	34 (13%)	43 (19%)	68 (27%)	
	Current	205 (76%)	174 (75%)	158 (62%)	
Number of comorbidities	0	163 (61%)	117 (51%)	137 (54%)	0.051**
	1	77 (29%)	68 (30%)	78 (30%)	
	≥ 2	28 (10%)	43 (19%)	41 (16%)	
BASDAI, median (IQR)		6.3 (4.8 to 7.4)	6.6 (5.2 to 7.9)	7.1 (5.5 to 7.9)	*0.001*
Spinal pain, median (IQR)		6.0 (4.0 to 8.0)	7.0 (5.0 to 8.0)	7.0 (5.0 to 8.0)	*0.002*
BASFI, median (IQR)		5.7 (3.7 to 7.6)	6.6 (4.8 to 8.3)	7.0 (5.1 to 8.6)	*< 0.001*
Chalder Fatigue Scale, median (IQR)		17.0 (14.0 to 21.0)	17.0 (13.0 to 21.0)	18.0 (13.0 to 22.0)	0.340
Remained on treatment		62 (23%)	49 (21%)	63 (25%)	0.670
Stopped treatment	Infection	20 (32%)	18 (37%)	15 (24%)	0.176
	Other adverse events	17 (27%)	15 (31%)	13 (21%)	
	Inefficacy or other reasons	25 (40%)	16 (33%)	33 (56%)	

Data presented as mean (standard deviation), median (interquartile range), number (percentage). Comparisons used *t*-test for continuous variables, chi-square test for categorical variables. Italicized text highlights significant differences. Examples of “other reasons” included patient choice and social circumstances

^+^HLA-B27 status available for 448 participants

*Above upper normal limit

**Non-parametric test for trend across ordered groups

*SD* standard deviation, *IQR* interquartile range, *mNY* modified New York criteria for Ankylosing Spondylitis, *BMI* body mass index, *BASDAI* Bath AS Disease Activity Index, *BASFI* Bath AS Functional Index

### All-cause TNFi discontinuation

Using marginal structural models, hazard ratios for all-cause TNFi discontinuation did not significantly differ in current (HR 0.79; 95%CI 0.53 to 1.20) or ex-smokers (HR 0.68; 95%CI 0.45 to 1.04), compared to never smokers. Unadjusted Kaplan-Meier estimators are shown in Additional file 1: Figure S2.

### Cause-specific TNFi discontinuation

Hazard ratios for each of the three causes of TNFi discontinuation according to smoking status are shown in Table 2. Our data did not provide evidence that current or previous smoking affected discontinuation due to infection, other adverse events or inefficacy/other reasons.

**Table 2:** All-cause and cause-specific treatment discontinuation according to baseline smoking status, after balancing differences in baseline covariates and accounting for those excluded from the analysis

	Never smoker	Ex-smoker	Current smoker
All-cause	Reference	0.68 (0.45 to 1.04)	0.79 (0.53 to 1.20)
Infection-related adverse events	Reference	1.03 (0.55 to 1.96)	0.79 (0.40 to 1.54)
Other adverse events	Reference	1.02 (0.50 to 2.07)	0.86 (0.41 to 1.78)
Inefficacy and other reasons	Reference	0.73 (0.39 to 1.37)	1.44 (0.86 to 2.41)

Results shown as hazard ratios (95% confidence interval). Examples of “other reasons” included patient choice and social circumstances

## Discussion

In this large UK cohort of axSpA participants, smoking was associated with significantly worse disease severity at initiation of TNFi therapy. However, TNFi discontinuation was not associated with baseline smoking status. Our results also did not provide evidence that smoking affected discontinuation due to infection-related or other adverse events. The results did suggest that current smokers may have higher risk of discontinuation due to inefficacy or other reasons.

The main strengths of this study are the quality of data and use of rigorous analyses. The BSRBR-AS cohort is representative of UK clinical practice, and its rich dataset allowed us to adjust for a large number of confounders. The use of inverse-probability weights allowed us to conduct improved time-to-event analysis for each cause of TNFi discontinuation (competing risk events), while accounting for baseline differences between exposure groups and time-varying disease activity within one model.

Our data did have some important limitations. While smoking has been recorded in detail, information on time since smoking cessation was not recorded. The proportion stopping due to adverse events was higher, while inefficacy was much lower, than reported elsewhere; nevertheless, the proportion of TNFi discontinuation was in keeping with existing literature [10]. In clinical practice, there is often an overlap between how inefficacy and adverse events are defined and reported. For instance, inflammation in previously unaffected peripheral joints may be reported as an adverse event, when it would more appropriately reflect lack of disease control and efficacy; therapy is more likely to continue in the face of mild adverse events if they are highly effective for symptom control. Before 2016—that is, the majority of this study period—the UK National Institute for Health and Care Excellence stipulated that patients who did not demonstrate initial or maintained response to treatment would not be funded to use a second TNFi [17, 18]. However, switching to a second TNFi was allowed if discontinuation was due to the development of a treatment-related adverse event. This may have influenced labelling of discontinuation as adverse events or other reasons. These errors would not affect analysis of all-cause discontinuation or discontinuation due to infection-related adverse events. It would have been interesting to examine the effect of changing smoking status at the individual level, but the number of such participants was too small to permit analysis. It is possible that TNFi stop dates in medical notes may be influenced by recall error; however, there is no evidence to suggest that such error differs according to smoking status, so we do not believe this will have introduced a bias. Lastly, the BSRBR-AS did not record pack-years to examine the effect of cumulative exposure. A significant effect is unlikely since none was found for ever vs never smokers.

There has been only one previous study of TNFi discontinuation and smoking in ankylosing spondylitis. Glintborg et al. reported increased risk of all-cause TNFi discontinuation in current and ex-smokers [4]. Compared to their study, our follow-up period was short. It is possible that differences in treatment persistence only becomes apparent with longer follow-up, although separation in Kaplan-Meier curves was clear by 2 years in their study [4]. Importantly, Glintborg et al. did not adjust for baseline differences in disease severity and BMI from their primary analysis, on account of the fact that these variables are potential mediators. Without adjusting for these known predictors of TNFi persistence [5, 19], it is difficult to attribute a causal effect of smoking on TNFi discontinuation. In DANBIO studies of psoriatic arthritis, significant differences in TNFi persistence were reported when analyses did not adjust for BMI and baseline disease severity [10], while analysis using essentially the same cohort but additionally adjusting for these covariates did not find such difference [20] (see Additional file 1: Table S4 for comparison).

Our negative results may have other explanations. The prevalence of current smoking was higher in DANBIO (43%) than in the BSRBR-AS (29%). Furthermore, we did not find smoking to be associated with age of symptom onset as previously reported [21]. It is possible that there are differences between the study cohorts. Our results are consistent with a growing body of evidence that smoking status does not independently impact TNFi treatment. In a study of obesity and TNFi response in axSpA, current smoking (as a covariate in their multivariable Cox regression model) was not associated with TNFi discontinuation (HR 0.92; 95%CI 0.66 to 1.28) [19]. Smoking status at TNFi initiation also did not affect treatment response in several studies [2, 19, 22–25], including a recent analysis of the BSRBR-AS [3]. These results should persuade clinicians to dispel any subconscious bias that smokers may not benefit as much from TNFi treatment.

We cannot exclude the possibility that smoking in early stages of the disease causes more severe disease observed at TNFi initiation. It is unknown whether smoking cessation improves disease severity irrespective of treatment. Smoking cessation remains a priority since patients with rheumatic diseases have high burdens of cardiovascular disease and the combined impact of TNFi and smoking on malignancy risk is unknown. Furthermore, smoking may be associated with greater radiographic progression [26].

## Conclusion

Smokers commencing TNFi were no more likely to discontinue treatment than never smokers in this large UK cohort of axSpA participants. To better estimate causal effects of smoking exposure, future studies would benefit from recording more detailed smoking history, and exploring cause-specific treatment discontinuation in larger cohorts with clearer recording of reasons for discontinuation.

## Additional file

Additional file 1: **Table S1.** Characteristics of the 840 patients exposed to TNFi and had smoking status, according to whether they were eligible for longitudinal analysis or were excluded. **Table S2.** Recoding mis-labelled discontinuation causes in the registry data. **Table S3.** Descriptions of stabilised inverse-probability weights used in analyses. **Table S4.** Comparing results from studies of TNFi persistence according to covariates used in their analysis models, particularly whether they have accounted for baseline disease severity. **Figure S1.** Standardised mean differences (SMD) for baseline variables before and after inverse-propensity weighting. **Figure S2.** Kaplan-Meier curves comparing all-cause discontinuation between smoking status. (DOCX 141 kb)

## Data Availability

BSRBR-AS data are held at the University of Aberdeen.
