# Supplementary material for: Smoking status and cause-specific discontinuation of tumour necrosis factor inhibitors in axial spondyloarthritis
Source: Arthritis Res Ther. 2019 Jul 22;21:177. doi: 10.1186/s13075-019-1958-z (PMC6647300; doi:10.1186/s13075-019-1958-z)
Supplement: Supplementary file 1 — Table S1. Characteristics of the 840 patients exposed to TNFi and had smoking status, according to whether they were eligible for longitudinal analysis or were excluded. Table S2. Recoding mis-labelled discontinuation causes in the registry data. Table S3. Descriptions of stabilised inverse-probability weights used in analyses. Table S4. Comparing results from studies of TNFi persistence according to covariates used in their analysis models, particularly whether they have accounted for baseline disease severity. Figure S1. Standardised mean differences (SMD) for baseline variables before and after inverse-propensity weighting. Figure S2. Kaplan-Meier curves comparing all-cause discontinuation between smoking status. (DOCX 141 kb) [file 13075_2019_1958_MOESM1_ESM.docx]

**Supplementary materials**

for “Smoking status and cause-specific discontinuation of tumour necrosis factor inhibitors in axial spondyloarthritis”

Sizheng Zhao, Kazuki Yoshida, Gareth T Jones, David M Hughes, Stephen J Duffield, Sara K Tedeschi, Houchen Lyu, Robert J Moots, Daniel H Solomon, Nicola J Goodson

Theoretical basis for using IPW approach

In our study, smoking exposure was simplified into smoking status at baseline. Studying the causal effect of baseline smoking status has conceptual difficulty, since we cannot randomly assign an individual to “having smoked for 20 years” at the onset of a hypothetical trial.

To help decide on the analytical approach, it is helpful to consider how causal effects *can* be estimated under ideal conditions. If the full smoking exposure history for each individual were known, it would be possible to “assign” smoking status at each time point – analogous to a sequentially randomised trial. (Descriptions of causal inference in observational studies often draw analogy from hypothetical randomised clinical trials. In contrast to conventional trials that are based on a single randomisation, sequentially randomised design allows the study of adaptive treatment strategies that adjust treatment in response to the observed course of disease.) Consider the following diagram (we omitted other variables for clarity):


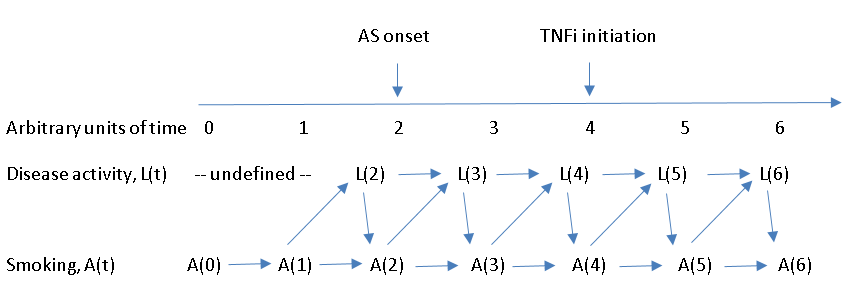


Where, $\bar{A}$(4) = smoking history until time 4

Current smoker: $\bar{A}$(4) = (a0, a1, a2, a3, 1)

Never smoker: $\bar{A}$(4) = (0, 0, 0, 0, 0)

Ex-smoker: $\bar{A}$(4) = (a0, a1, a2, a3, 0), where a0 + a1 + a2 + a3 ≥ 1 (smoked at some point)

A(4) = baseline smoking status

L(4) = baseline disease activity

Baseline smoking status, A(4), is confounded by $\bar{L}$(4) and $\bar{A}$(3), which are not available in most studies. Decision therefore rests on whether to control for baseline disease activity, L(4).

L(4) is a mediator with respect to $\bar{A}$(3)

L(4) is a confounder for A(4), and is a time-varying confounder that is affected by past smoking history, $\bar{A}$(3)

There are thus three options to approximate the effect of $\bar{A}$(4):

1. Do not account for L(4): entire $\bar{A}$(4) is confounded. See [1, 2].
2. Condition on L(4): A(4) unconfounded; $\bar{A}$(3) confounded; selection bias since L(4) is a collider. See reference [3].
3. Using IPW to control for L(4) without conditioning: A(4) unconfounded; $\bar{A}$(3) confounded.

Thus, we chose 3), which may be somewhat less problematic than 1) and 2).

Constructing inverse-probability weights

Stabilised IP “treatment” weights (IPTW, to balance baseline characteristics between smoking status) were constructed as follows: the numerator is predicted probability from a multinomial logistic model with smoking (A) as the only variable, and the denominator is the same model conditioned on covariates listed in the main text (L).

*IPW* = $\frac{Pr[A=a]}{Pr[A=a|L]}$

For each cause of TNFi discontinuation, the two other causes were censored. However, this renders unlikely a key assumption of time-to-event analysis: that the probability of being censored is the same for all individuals at risk; that is, censoring does not depend on baseline characteristics. We therefore used stabilised time-varying IP censoring weights (IPCW) to account for this dependent censoring. For analysis of time to adverse event, IPCWs for inefficacy and other reasons were multiplied and incorporated to the rest of the analysis.

Taking adverse events as the censoring event example, IPCW was constructed as follows:

*IPCW* =­­­ $\prod_{k=0}^{t} \frac{Pr[C\left( k+1 \right)=0|C\left( k \right)=0, \bar{A}\left( k \right), L (0)]}{Pr[C\left( k+1 \right)=0|C\left( k \right)=0, \bar{A}\left( k \right), \bar{L}\left( k \right)]}$

Where C(t) is a dichotomous variable taking the value 1 if a subject is censored in month t and 0 otherwise, $\bar{A}$(t) is the smoking history (in our case unchanged), L(0) are baseline covariates including disease severity variables, and $\bar{L}$(k) is the history of covariates (disease severity at baseline and before censoring). Time, as a restricted cubic spline (-mkspline- command in Stata v13), was also included in both numerator and denominator.

Participants excluded from the analysis set were represented by included participants with the same baseline smoking status and covariates. Stabilised baseline time-invariant IPCW were constructed as follows: the numerator is the predicted probability from logistic models of not being excluded (C=0) conditioned on smoking status (A), over the same model additionally conditioned on covariates (L).

*IPCW* = $\frac{Pr[C=0|A]}{Pr[C=0|L,A]}$

All weights were multiplied before use in pooled logistic models.

Multiple imputation

To generate the above weights, (multinomial/) logistic models required complete data for all covariates. Multiple imputation was performed using chained equations (-mi impute mice- command in Stata v13). All variables in each IPW model were included in the respective imputation models, with 30 imputed datasets. Logistic (ordinal/multinomial) models were used for categorical variables and predictive mean matching for continuous variables, which accounts for their restricted range.

**Results**

| Supplementary Table S1. Characteristics of the 840 patients exposed to TNFi and had smoking status, according to whether they were eligible for longitudinal analysis or were excluded. | | | | | | |
| --- | --- | --- | --- | --- | --- | --- |
|  | | | Included (n=758) | Excluded (n=82) | P-value |  |
| Discontinued treatment | | | 174 (23%) | 14 (17%) | 0.220 |  |
| Smoking status | | Never | 271 (36%) | 44 (54%) | 0.006 |  |
|  |  | Ex | 231 (30%) | 19 (23%) |  |  |
|  |  | Current | 256 (34%) | 19 (23%) |  |  |
| Age, mean (SD) years | | | 45.0 (13.5) | 46.9 (13.8) | 0.230 |  |
| Male | | | 504 (66%) | 62 (76%) | 0.094 |  |
| Meets mNY criteria for AS | | | 480 (63%) | 57 (70%) | 0.270 |  |
| HLA-B27 positive | | | 416 (75%) | 53 (82%) | 0.270 |  |
| Elevated CRP* | | | 440 (61%) | 47 (60%) | 0.890 |  |
| Age at symptom onset, median (IQR) years | | | 26.0 (20.0, 33.0) | 23.0 (19.0, 31.0) | 0.028 |  |
| Symptom duration, median (IQR) years | | | 14.8 (5.7, 27.6) | 19.3 (8.4, 30.8) | 0.020 |  |
| BMI, mean (SD) | | | 28.0 (5.7) | 27.2 (5.3) | 0.240 |  |
| Quintiles of Index of Multiple Deprivation | 1, most deprived | | 157 (21%) | 10 (12%) | <0.001** |  |
|  | 2 | | 127 (17%) | 16 (20%) |  |  |
|  | 3 | | 156 (21%) | 10 (12%) |  |  |
|  | 4 | | 171 (23%) | 27 (33%) |  |  |
|  | 5, most affluent | | 147 (19%) | 19 (23%) |  |  |
| Highest level of education | Secondary school | | 261 (35%) | 29 (36%) | 0.810 |  |
|  | Apprenticeship | | 71 (9%) | 7 (9%) |  |  |
|  | Further education college | | 235 (31%) | 21 (26%) |  |  |
|  | University degree | | 137 (18%) | 18 (22%) |  |  |
|  | Further degree | | 46 (6%) | 6 (7%) |  |  |
| Alcohol status | Never | | 75 (10%) | 4 (5%) | 0.280 |  |
|  | Ex | | 145 (19%) | 14 (17%) |  |  |
|  | Current | | 537 (71%) | 63 (78%) |  |  |
| Number of comorbidities | 0 | | 417 (55%) | 51 (63%) | 0.025** |  |
|  | 1 | | 223 (30%) | 21 (26%) |  |  |
|  | ≥2 | | 112 (15%) | 9 (11%) |  |  |
| Data presented as mean (standard deviation), median (interquartile range), number (percentage). Comparisons used t-test for continuous variables, Chi-squared test for categorical variables. Bold text highlights significant differences.  *Above upper normal limit.  **Non-parametric test for trend across ordered groups.  SD, standard deviation; IQR, interquartile range; mNY, modified New York criteria for Ankylosing Spondylitis; BMI, body mass index; BASDAI, Bath AS disease activity index; BASFI, Bath AS functional index. | | | | | | |

| Supplementary Table S2. Recoding mis-labelled discontinuation causes in the registry data. | | | | | | |
| --- | --- | --- | --- | --- | --- | --- |
|  | | | | Before correction | | After correction |
| Continued TNFi | | | | 584 | 584 | |
| Discontinued TNFi due to: | **Adverse events** | | Infection | 62 | | 45 |
|  |  |  | Others |  |  | 53 |
|  | **Inefficacy** | | | 7 | | 10 |
|  | **Symptom in remission** | Infection | | 18 | | - |
|  |  | Other adverse events | | 6 | | - |
|  |  | Consultant decision | | 7 | | - |
|  |  | Patient decision | | 1 | | - |
|  |  | Other | | 15 | | - |
|  | **Other** | | | 58 | | 66 |
| Data were entered into “symptom in remission” category with accompanying free-text explanations. Adverse events, inefficacy and other reasons were regrouped into their respective categories. Consultant decisions were grouped into inefficacy, and patient decision into other. | | | | | | |


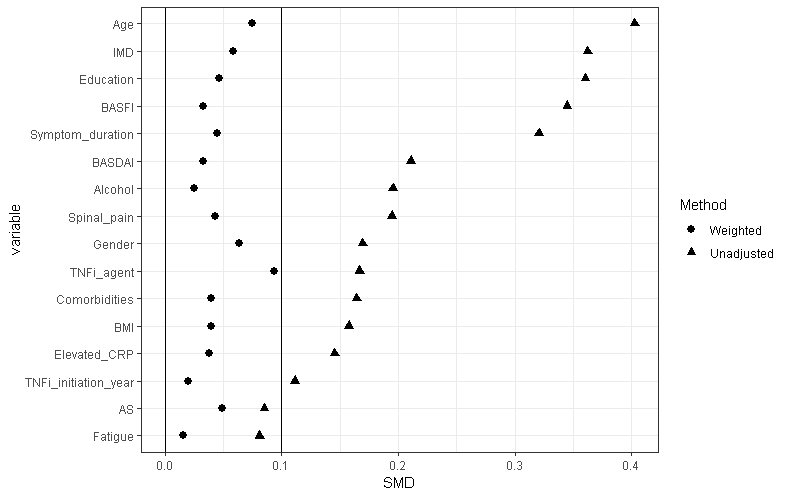


Supplementary Figure S1. Standardised mean differences (SMD) for baseline variables before and after inverse-propensity weighting. SMD<0.1 has been taken to indicate negligible difference. BASDAI, Bath AS disease activity index; BASFI, Bath AS functional index; CFQ, Chalder Fatigue Scale; IMD, Index of Multiple Deprivation.


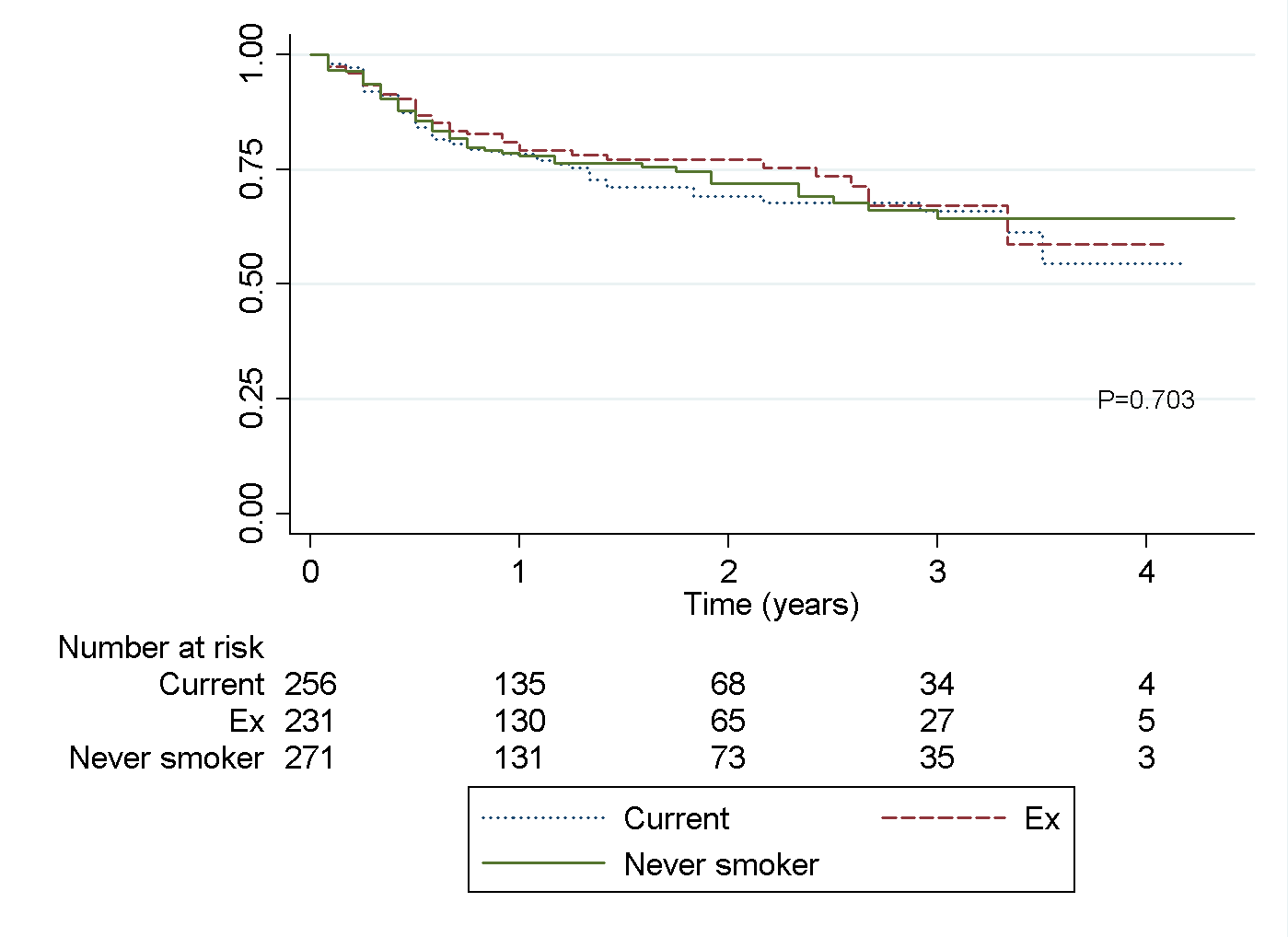


Supplementary Figure S2. Kaplan-Meier curves comparing all-cause discontinuation between smoking status. P-value from the log-rank test of equality.

| Supplementary Table S3. Descriptions of stabilised inverse-probability weights used in analyses. | | | | | | |
| --- | --- | --- | --- | --- | --- | --- |
|  | | Mean | SD | Min | Max |  |
| Time invariant weights | IPTW to balance baseline covariates between smoking status | 0.98 | 0.53 | 0.40 | 4.66 |  |
|  | IPCW to account for excluded subjects | 1.00 | 0.13 | 0.89 | 3.27 |  |
| Time-varying weights for dependent censoring | Censoring | 1.00 | 0.04 | 0.76 | 1.82 |  |
|  | Adverse events | 1.00 | 0.14 | 0.41 | 5.25 |  |
|  | Inefficacy | 1.00 | 0.13 | 0.47 | 4.54 |  |
|  | Other | 1.00 | 0.09 | 0.49 | 2.87 |  |
| IPTW, inverse-probability of “treatment” weights; IPCW, IP censoring weights; SD standard deviation. | | | | | | |

| Supplementary Table S4. Comparing results from studies of TNFi persistence according to covariates used in their analysis models, particularly whether they have accounted for baseline disease severity. | | | | |
| --- | --- | --- | --- | --- |
|  | Cohort and sample size | Variables in Cox models | Effect size for smoking (Hazard ratio; 95% confidence interval) |  |
| Axial spondyloarthritis | BSRBR-AS  N=598  802 patient years | Age, gender, symptom duration, education, classification as AS, deprivation, comorbidity, TNFi agent, year of TNFi initiation;  elevated CRP, BMI, BASAI, spinal pain, BASFI, fatigue. | Current v never: 0.79 (0.53 to 1.20)  Ex- v never: 0.68 (0.45 to 1.04) |  |
|  | Swiss Clinical Quality Management cohort [4]  N=343 | Age, gender, HLA-B27, classification as AS, exercise;  BASDAI, BASMI, elevated CRP, BMI. | Current vs non-current: 0.92 (0.66 to 1.28) |  |
|  | DANBIO [1]  N=1425  5983 patient years | Age, gender, disease duration, year of TNFi initiation. | Current v never:  1.41 (1.21 to 1.65)  Ex- v never: 1.38 (1.12 to 1.65) |  |
| Psoriatic arthritis | DANBIO [2]  N=1148  2790 patient years | Age, disease duration, year of TNFi initiation, swollen joint. | Current vs never smoking:  Etanercept: 1.74 (1.14 to 2.66)  Infliximab: 1.62 (1.06 to 2.48)  Adalimumab: 0.80 (0.52 to 1.23) |  |
|  | DANBIO/ICEBIO [5]  N=1271 (approximately 10% ICEBIO)  5142 patient years | Age, gender, disease duration, methotrexate use, year of TNFi initiation, nationality (Denmark/Iceland);  pain, HAQ, DAS28, CRP. | Current vs non-current smoking: 1.20 (0.96 to 1.50) |  |
| AS, ankylosing spondylitis; BASDAI, Bath AS disease activity index; BASFI, Bath AS functional index; BASMI, Bath AS metrology index; DAS28, Disease activity score – 28 joint count; HAQ, health assessment questionnaire; TNFi, TNF inhibitor. | | | |  |

**References**

1. Glintborg B, Hojgaard P, Lund Hetland M, Steen Krogh N, Kollerup G, Jensen J, Chrysidis S, Jensen Hansen IM, Holland-Fischer M, Hojland Hansen T *et al*: **Impact of tobacco smoking on response to tumour necrosis factor-alpha inhibitor treatment in patients with ankylosing spondylitis: results from the Danish nationwide DANBIO registry**. *Rheumatology* 2016, **55**(4):659-668.

2. Hojgaard P, Glintborg B, Hetland ML, Hansen TH, Lage-Hansen PR, Petersen MH, Holland-Fischer M, Nilsson C, Loft AG, Andersen BN *et al*: **Association between tobacco smoking and response to tumour necrosis factor alpha inhibitor treatment in psoriatic arthritis: results from the DANBIO registry**. *Annals of the rheumatic diseases* 2015, **74**(12):2130-2136.

3. Ciurea A, Scherer A, Weber U, Exer P, Bernhard J, Tamborrini G, Riek M, Muller RB, Weiss B, Nissen MJ *et al*: **Impaired response to treatment with tumour necrosis factor alpha inhibitors in smokers with axial spondyloarthritis**. *Annals of the rheumatic diseases* 2016, **75**(3):532-539.

4. Micheroli R, Hebeisen M, Wildi LM, Exer P, Tamborrini G, Bernhard J, Moller B, Zufferey P, Nissen MJ, Scherer A *et al*: **Impact of obesity on the response to tumor necrosis factor inhibitors in axial spondyloarthritis**. *Arthritis research & therapy* 2017, **19**(1):164.

5. Hojgaard P, Glintborg B, Kristensen LE, Gudbjornsson B, Love TJ, Dreyer L: **The influence of obesity on response to tumour necrosis factor-alpha inhibitors in psoriatic arthritis: results from the DANBIO and ICEBIO registries**. *Rheumatology* 2016, **55**(12):2191-2199.
